# Dataset on psychological determinants of impulsive buying in Vietnamese social commerce

**DOI:** 10.1016/j.dib.2026.113080

**Published:** 2026-07-15

**Authors:** Lan Van Thi Mai, Hai Ninh Nguyen

**Affiliations:** Foreign Trade University, Hanoi, Vietnam

**Keywords:** Impulsive buying behavior, Fear of missing out, Immediate gratification, Hedonic motivation, Perceived social value, Reflective measurement, Vietnam

## Abstract

This article presents a survey dataset on psychological drivers and impulsive buying behavior on social commerce platforms in Vietnam. The data were collected between January and April 2026 through a cross-sectional online questionnaire administered to Vietnamese consumers who had purchased at least one product through a social commerce platform within the previous six months. The refined analytical dataset contains 589 valid observations retained after removal of 42 submitted cases that showed straight-lining, duplicate response patterns, or other survey-quality problems from an initial pool of 631 responses. The instrument contains 31 Likert-type indicators measuring eight reflective constructs: perceived enjoyment, hedonic motivation, stress state, social influence, immediate gratification, perceived social value, fear of missing out, and impulsive buying behavior. The repository package contains the cleaned dataset, codebook, questionnaire, informed consent materials, and repository documentation. The repository package also reports reflective measurement-model diagnostics computed in SmartPLS 4, including indicator loadings, reliability coefficients, average variance extracted, heterotrait–monotrait ratios, and collinearity statistics, so that users can inspect measurement properties before reuse. These data can be reused for scale assessment, replication, multigroup comparison, mediation and moderation analysis, and comparative research on social commerce and digital consumer behavior in emerging-market contexts.

Specifications Table

**Table:** 

Subject	Social Sciences
Specific subject area	Social commerce, digital consumer behavior, impulse buying, and online consumer psychology.
Type of data	Table; survey dataset; codebook; questionnaire; informed consent form.
Data collection	Structured online questionnaire administered through Google Forms and distributed to Vietnamese consumers with recent purchase experience on social commerce platforms using non-probability purposive sampling. Respondents provided informed consent and passed one eligibility screening question. Of 631 submitted responses, 589 were retained in the cleaned dataset after exclusion of 42 cases showing straight-lining, duplicate response patterns, or other survey-quality problems. Questionnaire items were adapted from established scales in digital consumer behavior and impulse-buying research.
Data source location	Vietnam.
Data accessibility	Repository: Mendeley Data. Dataset title: Survey dataset on psychological drivers of impulsive buying behavior on social commerce platforms among Vietnamese consumers. Repository name: Mendeley Data Data identification number: 10.17632/9kb3djy4r5.1 Direct URL to data: https://doi.org/10.17632/9kb3djy4r5.1 Instructions for accessing these data: direct download via the Mendeley Data DOI link above; the repository is openly accessible without registration.
Related research article	None.

## 1 Value of the Data

• This dataset provides consumer-level survey data from Vietnam, an emerging market where social commerce combines social interaction, livestream shopping, platform-based recommendations, and direct purchasing within the same digital environment.
• The dataset is useful for researchers examining impulsive buying behavior in social commerce because it includes affective, social, motivational, and situational constructs measured within a common survey instrument.
• The data can be reused for scale assessment, measurement-model evaluation, multigroup analysis, mediation and moderation analysis, and comparisons across consumer groups or preferred social commerce platforms.
• The dataset can support comparative research across countries and market contexts, particularly studies examining whether psychological drivers of impulsive buying differ between emerging markets and more mature digital commerce environments.

## 2 Background

Social commerce has become an increasingly important part of the digital marketplace, where shopping activities are embedded in platform environments that combine social interaction and transactional exchange [1]. Through comments, reviews, livestreams, peer recommendations, and visible purchase cues, consumers can discover, evaluate, discuss, and purchase products within the same social-media-based setting. Recent marketing and information-systems literature therefore treats social commerce as a distinctive domain of digital consumer behavior rather than as a simple extension of conventional e-commerce [1,2]. At the same time, impulsive buying behavior continues to attract attention because digitally mediated environments can shorten evaluation time, intensify emotional arousal, and increase exposure to social and promotional stimuli that trigger unplanned purchases [2,3], which makes them a relevant context for documenting reusable consumer-level survey data.

Prior literature indicates that impulsive buying in online and social commerce settings is associated with multiple psychological mechanisms rather than with a single determinant. Positive experiential responses such as perceived enjoyment and hedonic motivation have been linked to shopping-related pleasure and entertainment value in technology-mediated environments [4,5]. Immediate gratification captures the short-term reward experienced when consumers make spontaneous purchases, while online impulse-buying research also highlights the role of affective responses and platform-related cues in shaping unplanned purchase tendencies [6,7]. Social commerce also makes social influence and perceived social value salient because consumers encounter reviews, recommendations, visible interactions, and socially meaningful consumption signals within the same platform environment [7,8]. Fear of missing out has also become relevant to digital shopping contexts characterized by urgency, limited offers, and concern about missing attractive opportunities experienced by others [3,9], complementing earlier consumer research on normative influences that shape impulsive buying behavior [10]. Stress-related emotional states have been used in parallel to document affective conditions relevant to consumer behavior in high-stimulation environments [11].

Building on this literature, the present dataset documents affective, social, and situational drivers of impulsive buying within a single social commerce instrument administered in Vietnam. Vietnam provides a relevant setting because platforms such as Facebook, TikTok Shop, Instagram, Zalo, and Shopee Live are increasingly embedded in everyday consumer activity, and the dataset captures purchase experience across these environments using a common reflective measurement structure [2,3,7,9]. The repository package combines the cleaned analytical dataset with the codebook, questionnaire, informed consent materials, and repository documentation, which together allow scale assessment, replication, and comparative analysis across constructs, platforms, and respondent subgroups.

## 3 Data Description

The repository package comprises five files that together document the analytical dataset and its accompanying metadata: Cleaned dataset.xlsx, Codebook.docx, Questionnaire_EN.docx, Informed Consent letter.docx, and README.docx. The file Cleaned dataset.xlsx contains the final analytical dataset with 589 valid observations retained after questionnaire screening and data-quality refinement. Each row represents one respondent and each column represents one analytical variable, resulting in a total of 38 variables, including seven demographic and behavioral variables and 31 measurement indicators covering eight reflective constructs, all measured on a five-point Likert scale ranging from 1 (strongly disagree) to 5 (strongly agree).

Codebook.docx documents variable names, labels, coding rules, valid values, and measurement levels for the measurement and demographic variables, together with the consent and screening variables used during questionnaire administration. Questionnaire_EN.docx provides the English-language survey instrument, comprising the cover text, consent statement, one eligibility screening question, the 31-item measurement block, and the demographic section. Informed Consent letter.docx records the information given to respondents concerning voluntary participation, anonymity, academic use of anonymized responses, and repository archiving, while README.docx summarizes dataset scope, structure, sample size, cleaning rules, and recommended reuse conditions.

Table 1 summarizes the operationalization of the eight reflective constructs used in the questionnaire. Table 2 reports the characteristics of the 589 respondents retained in the cleaned analytical dataset. Female respondents account for 64.01% of the sample and male respondents for 31.07%. Respondents aged 25–34 years (41.09%) and 18–24 years (30.05%) form the two largest age groups. Bachelor’s degree holders account for the largest educational share (53.99%). Shopee Live / Shopee social features (32.09%) and TikTok Shop (31.07%) are the two most frequently reported preferred platforms, and fashion and accessories (29.03%) and cosmetics and skincare (23.94%) are the two most frequently purchased product categories.

**Table 1:** Measurement constructs.

Construct	Code	Statement	Sources
Perceived Enjoyment	PE1	Using social commerce platforms for shopping is enjoyable.	[4,7]
	PE2	I find shopping on social commerce platforms fun.	
	PE3	I feel pleased when browsing products on social commerce platforms.	
	PE4	Shopping on social commerce platforms is entertaining.	
Hedonic Motivation	HM1	I shop on social commerce platforms because it is enjoyable.	[5]
	HM2	I shop on social commerce platforms because it is exciting.	
	HM3	I shop on social commerce platforms because it is entertaining.	
	HM4	I shop on social commerce platforms because it makes me feel good.	
Stress State	SS1	When browsing social commerce platforms, I feel tense.	[11]
	SS2	When shopping on social commerce platforms, I feel stressed.	
	SS3	I feel emotionally pressured to make quick purchase decisions on social commerce platforms.	
	SS4	I feel overwhelmed by the amount of shopping information and offers on social commerce platforms.	
Social Influence	SI1	People who are important to me think that I should shop through social commerce platforms.	[5,7]
	SI2	People who influence my behavior encourage me to buy through social commerce platforms.	
	SI3	Reviews, comments, and live interactions from others affect my purchase decisions on social commerce platforms.	
	SI4	I tend to follow other people’s buying suggestions on social commerce platforms.	
Immediate Gratification	IG1	Buying impulsively on social commerce platforms gives me immediate enjoyment.	[6,10]
	IG2	I feel pleased when I make an impulsive purchase on a social commerce platform.	
	IG3	Impulsive purchases on social commerce platforms are exciting to me.	
	IG4	Buying immediately on social commerce platforms gives me instant satisfaction.	
Perceived Social Value	PSV1	Buying on social commerce platforms improves the way I am perceived by others.	[8]
	PSV2	My purchases on social commerce platforms can help me gain social approval.	
	PSV3	Buying on social commerce platforms makes me feel socially accepted.	
	PSV4	My purchases on social commerce platforms can create a good impression on others.	
Fear of Missing Out	FOMO1	I worry that I may miss attractive deals or offers on social commerce platforms.	[9]
	FOMO2	I feel uneasy when other people get product information or deals before I do on social commerce platforms.	
	FOMO3	I am afraid of missing important shopping opportunities on social commerce platforms.	
Impulsive Buying Behavior	IB1	I often buy products on social commerce platforms spontaneously.	[7,10]
	IB2	I sometimes buy products on social commerce platforms without planning in advance.	
	IB3	I make purchase decisions quickly when shopping on social commerce platforms.	
	IB4	I buy things on social commerce platforms on the spur of the moment.

**Table 2:** Sample characteristics (n = 589).

Variable	Category	Frequency	Percentage (%)
Gender	Male	183	31.07
	Female	377	64.01
	Prefer not to say / Other	29	4.92
Age group	18–24	177	30.05
	25–34	242	41.09
	35–44	118	20.03
	45+	52	8.83
Highest education level	High school or below	71	12.05
	College diploma	112	19.02
	Bachelor’s degree	318	53.99
	Postgraduate	88	14.94
Monthly income	≤ 10 million VND	259	43.97
	10–20 million VND	224	38.03
	> 20 million VND	106	18.00
Most preferred social commerce platform	Facebook	141	23.94
	TikTok Shop	183	31.07
	Instagram	41	6.96
	Zalo	35	5.94
	Shopee Live / Shopee social features	189	32.09
Purchase frequency on social commerce	Less than once a month	94	15.96
	1–2 times/month	218	37.01
	3–4 times/month	165	28.01
	5+ times/month	112	19.02
Most frequently purchased product category	Fashion & accessories	171	29.03
	Cosmetics & skincare	141	23.94
	Household items	82	13.92
	Food & beverages	77	13.07
	Electronics & gadgets	59	10.02
	Mother & baby products	41	6.96
	Books / stationery / gifts	18	3.06

## 4 Experimental Design, Materials and Methods

The data were collected through a cross-sectional online survey conducted between January and April 2026 using a non-probability purposive sampling approach. The target population comprised Vietnamese consumers who had purchased at least one product through a social commerce platform, such as Facebook, TikTok Shop, Instagram, Zalo, or Shopee Live, within the previous six months. This sampling approach was appropriate because the study specifically targeted respondents with recent and relevant social commerce purchasing experience rather than the general adult population. The questionnaire was administered online using Google Forms and distributed digitally to eligible participants across Vietnam.

The questionnaire began with an information sheet and an electronic consent statement. Respondents were informed about the purpose of the questionnaire, voluntary participation, approximate completion time, anonymity protections, and the academic use and repository archiving of anonymized responses. Only those who agreed to participate proceeded to the eligibility screening section, which used a single question asking whether the respondent had purchased at least one product through a social commerce platform during the previous six months. Respondents who answered “No” were screened out before the measurement section.

A total of 631 responses were submitted. After initial review, 42 cases were removed because they exhibited invalid response patterns, including straight-lining, duplicate response patterns, and other survey-quality problems, leaving a cleaned analytical dataset of 589 valid observations. The analytical file released in the repository contains only the final retained cases and therefore excludes the consent and screening variables. The substantive measurement block contains 31 indicators across eight reflective constructs measured on a five-point Likert scale: perceived enjoyment (4 items), hedonic motivation (4 items), stress state (4 items), social influence (4 items), immediate gratification (4 items), perceived social value (4 items), fear of missing out (3 items), and impulsive buying behavior (4 items), each adapted from established scales in online consumer behavior, technology use, perceived value, fear of missing out, affective state measurement, and impulse-buying research.

Measurement quality was assessed in SmartPLS 4, restricted to the reflective measurement model in line with the scope of a Data in Brief article [12]. Cronbach’s alpha values ranged from 0.745 to 0.857, rho_A values ranged from 0.747 to 0.869, and composite reliability values ranged from 0.839 to 0.904. All average variance extracted values exceeded 0.50, ranging from 0.566 to 0.702. Indicator loadings ranged from 0.731 to 0.927, indicating acceptable indicator reliability and convergent validity for all constructs, as reported in Table 3. Table 4 reports the heterotrait–monotrait ratios for discriminant validity assessment. All HTMT values are below 0.85, with the highest value observed between social influence and perceived social value (0.806), indicating acceptable discriminant validity [13]. Collinearity diagnostics were also inspected, and the variance inflation factor values ranged from 1.061 to 2.016, indicating no serious multicollinearity problems.

**Table 3:** Reliability and convergent validity.

Construct	Indicator loadings	Cronbach’s alpha	rho_A	Composite reliability	AVE
FOMO	0.791–0.867	0.774	0.815	0.866	0.683
HM	0.734–0.800	0.773	0.776	0.854	0.595
IB	0.778–0.867	0.844	0.845	0.895	0.682
IG	0.731–0.816	0.795	0.801	0.867	0.620
PE	0.761–0.815	0.791	0.793	0.864	0.615
PSV	0.778–0.838	0.838	0.839	0.891	0.673
SI	0.743–0.927	0.857	0.869	0.904	0.702
SS	0.734–0.772	0.745	0.747	0.839	0.566

**Table 4:** Discriminant validity using HTMT.

	FOMO	HM	IB	IG	PE	PSV	SI	SS
FOMO								
HM	0.232							
IB	0.326	0.578						
IG	0.345	0.730	0.770					
PE	0.284	0.491	0.601	0.782				
PSV	0.318	0.253	0.616	0.504	0.267			
SI	0.345	0.298	0.590	0.511	0.278	0.806		
SS	0.208	0.185	0.416	0.517	0.254	0.179	0.207	

## Limitations

Several limitations of the dataset should be noted. The data were collected through a cross-sectional online survey using non-probability purposive sampling, so the retained sample should not be interpreted as statistically representative of the wider Vietnamese population. Because the variables are based on self-reported responses and on respondents’ recollection of recent shopping experiences in such environments, the dataset may carry recall bias and common-method bias. The released analytical file contains only valid retained cases and therefore does not allow direct inspection of excluded responses within the same spreadsheet, and the data reflect one national context and one period of collection, so responses may vary across countries, periods, or platform environments.

## Ethics Statement

This work involved human participants in an anonymous online questionnaire. Before accessing the questionnaire, respondents were presented with an information and consent page describing the purpose of the questionnaire, approximate completion time, voluntary participation, confidentiality protections, and the intended academic use of the data. Only those who provided electronic informed consent were allowed to proceed to the screening question and the main questionnaire.

Participants were informed that no directly identifiable personal information, such as full name, phone number, or personal email address, would be collected. All responses were recorded anonymously and handled confidentially. The consent materials stated that anonymized responses may be used for academic research purposes, including dissertations, journal articles, conference papers, research reports, and archiving in an open research data repository for transparency, verification, and reuse.

Participation was entirely voluntary, and respondents could discontinue the questionnaire at any time before submission without penalty. Because the data were collected through an anonymous, non-invasive questionnaire and no sensitive personal identifiers were collected, formal ethics committee approval was not required under applicable institutional regulations. The questionnaire was conducted in accordance with the ethical principles of the Declaration of Helsinki.

## CRediT Author Statement

**Mai Thi Lan Van**: Conceptualization, Methodology, Investigation, Data collection, Data curation, Formal analysis, Writing - original draft. **Hai Ninh Nguyen**: Supervision, Methodological validation, Writing - review and editing, Correspondence.

## Data Availability

Mendeley DataSurvey dataset on psychological drivers of impulsive buying behavior on social commerce platforms among Vietnamese consumers (Original data).
